# Decimeter-Level Geolocation Accuracy Updated by a Parametric Tropospheric Model with GF-3

**DOI:** 10.3390/s18072197

**Published:** 2018-07-08

**Authors:** Wentao Wang, Jiayin Liu, Xiaolan Qiu

**Affiliations:** 1 School of Electronic, Electrical and Communication Engineering, University of Chinese Academy of Sciences, Beijing 101408, China; wangwentao16@mails.ucas.ac.cn; 2 Institute of Electronics, Chinese Academy of Sciences, Beijing 100190, China; xlqiu@mail.ie.ac.cn; 3 Key Laboratory of Technology in Geo-spatial Information Processing and Application System, Beijing 100190, China

**Keywords:** GF-3, SAR, geolocation accuracy, tropospheric model

## Abstract

GaoFen-3 (GF-3) is a multi-polarization C-band synthetic aperture radar (SAR) satellite in China with a resolution of up to 1 m. Up to now, the geolocation accuracy of GF-3 could be improved to 3 m. According to the current study, there still exist meter-level geolocation residuals caused by atmospheric path delay after compensating with a static tropospheric model. In this paper, we compensate the residuals with the sophisticated tropospheric model based on real meteorological data. The experimental results show that the tropospheric model has an accuracy on the millimeter level, which can increase GF-3’s geolocation accuracy to several decimeters compared with the static tropospheric model.

## 1. Introduction

In the past twenty years, a great success was achieved in the development of Chinese spaceborne synthetic aperture radar (SAR) technique. Not only the SAR imaging instruments, but the post-processing systems all reach an advanced level comparable to the other state-of-the-art technology in the world. The GaoFen-3 (GF-3) satellite, one of Earth’s observation satellites included in China’s high-resolution satellite scheme, launched in August of 2016. It is the first high-resolution C-band (5.4 GHz) SAR in China, carrying a state-of-the-art SAR imaging instrument. It operates on low earth orbit at 755 km and works in 12 different imaging modes [1,2]. With its ability to work in all-weather and day-and-night conditions, GF-3 will play an important role in the field of ocean monitoring, resource detection, disaster prevention, and emergency rescuing.

With the advances in spaceborne SAR imaging technique, the resolution of GF-3’s imaging is up to 1 m. However, the geolocation accuracy of GF-3 cannot reach the same level, which will cause a mismatch in the SAR image registration and inappropriate image mosaics. At present, the geolocation accuracy of GF-3 is within 3 m [3], and there is still an amount of work to be done compared with the state-of-the-art SAR system’s in the world, such as TerraSAR-X in Germany, which can achieve an accuracy of a sub-meter [4,5]. In order to improve the accuracy of GF-3 to better than 1 m, a more detailed scheme needs to be designed. There are still many error sources in the procedure of GF-3’s geolocation: orbit determination accuracy, atmospheric path delay, system delay, solid Earth tides (SET), and the precise position of corner reflectors [3]. Among them, the atmospheric model used in GF-3 currently causes meter-level residuals, which limit the geolocation accuracy of GF-3. Thus, in this paper, the emphasis is put on the correction of the atmospheric path delay.

The atmospheric model was initially used in the correction of the radio wave delay. Then, it was widely used for Global Positioning System (GPS) and other satellite navigation systems [6,7]. Recently, many scholars and institutions have applied the atmospheric model to the procedure of spaceborne SAR geolocation. The influence of the atmosphere on geolocation is mainly concentrated on the troposphere and ionosphere. For the correction of the tropospheric delay, the German Aerospace Center (DLR) used a simplified static tropospheric model for all geolocation and geocoding and annotated it in the TerraSAR-X SAR products [8,9,10]. Schubert [11] and Deng [12] replaced the annotated path delay with a height-dependent model, which is based on surface meteorological data, in the process of the TerraSAR-X and YaoGan products. Cong [13] and Nitti [14] compensated the path delay with a refined atmospheric model, which is called the raytracing model or integral model. It makes use of numerical weather data and achieves mm accuracy. A comprehensive discussion concerning the effects and accuracy of the abovementioned models can be found in [15]. As for the ionospheric model, a detailed introduction and analysis used for GPS can be found in [6]. A simplified model is used in spaceborne SAR geolocation to correct the ionospheric delay [10,12,15,16]. Vertical total electron content (VTEC) used in the model can be acquired from dedicated websites. Meyer et al. [17] provide an in-depth study of the ionospheric total electron content (TEC) of the L-band SAR system.

At present, the tropospheric model used in GF-3’s absolute geolocation procedure is the same as that in the TerraSAR-X simplified model, which has a poor accuracy. A meter-level geolocation residual will be introduced to the results for the lack of the support of meteorological data in the simplified static model. In this paper, we analyze the accuracy of different tropospheric models and compensate the tropospheric path delay in GF-3 with the integral model. The experiments verified that the tropospheric model used in this paper achieves the highest accuracy among all models. The real zenith tropospheric path delays acquired from the International Global Navigation Satellite System (GNSS) Service (IGS) stations prove the results. The updated atmospheric path delay can be improved by several decimeters in slant range, in contrast with the simplified static model. In the following, Section 2 gives a brief introduction of several atmospheric models. Two experiments are carried out in Section 3. Section 3.1 analyzes the accuracy of different tropospheric models, and Section 3.2 applies the tropospheric models to the geolocation of GF-3. Our conclusions are described in Section 4.

## 2. Atmospheric Path Delay

According to the various characteristics of the atmosphere in the vertical direction, the atmosphere can be divided into several different parts. For the path delay, the atmosphere is mainly divided into two parts: neutral atmosphere and ionosphere [18]. The ionosphere is the upper atmosphere ionized by the excitation of solar energetic radiation and cosmic rays. The neutral atmosphere refers to the atmosphere that contains the troposphere, stratosphere, middle layer, and part of the thermosphere. Among them, the troposphere has the greatest influence on the delay of the signal transmission, so the signal delay happening in the neutral atmosphere is generally called the tropospheric delay. The tropospheric path delay is nondispersive, that is, the refractive index is independent of the frequency of the signal and only relates to the propagation speed. However, the ionosphere delay is dispersive, which is related to the frequency of the signal, and the ionospheric group delay is of interest to the SAR signal.

There will be two main effects for the SAR signal caused by the atmosphere: (1) the atmospheric refractive index is varied with the density of the atmosphere at different heights, so the refraction of the signal causes the propagation path of the SAR signal to be bent; and (2) The medium in the atmosphere leads to a slower propagation speed of electromagnetic waves in the atmosphere than the velocity in the vacuum, and the delay can be equivalent to the extension of the signal propagation path.

**Figure 1:**
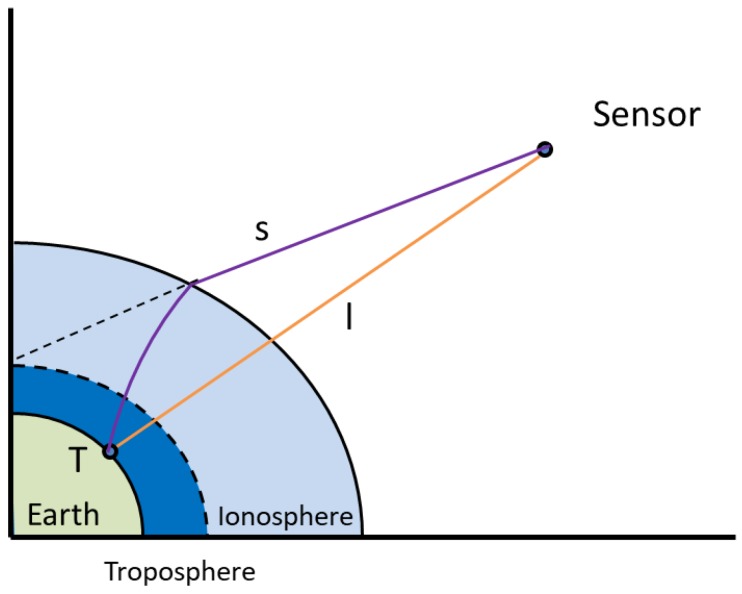
The propagation path of the signal in the atmosphere (disproportional schematic scheme).

Figure 1 illustrates the difference between the actual propagation path (purple) and the ideal propagation path (yellow) when the SAR signal propagates into the atmosphere. The difference in the distance ΔL is related to the atmospheric refractive index n(s) and is written as follows:

(1) $$\begin{array}{c} \Delta L=\int_{s}n(s)ds-\int_{l}dl\\ \;\;\;\;\;\;\;\;\;\;\;\;\;\;\;\;\;\;\;\;\;\;\;\;\;\;\;\;\;\;\;\;\;\;=\int_{s}\left(n(s)-l)ds+(\int_{s}ds-\int_{l}dl\right) \end{array}$$

where s and l represent the actual propagation path and the geometric straight path, respectively. There are mainly two solutions to Equation (1): one is the series expansion method, and the other is the mapping function method. The later method is more commonly used, and it expresses Equation (1) in the form of the product of the zenith delay ΔZ and the projection function MF(θ) as follows:

(2) ΔL=ΔZ⋅MF(θ).

Generally, the incidence angle θ of GF-3 is between 10° and 60°. A simple projection function is used as follows:

(3) $$MF(\theta )=\frac{1}{\cos\theta }$$

The following parts provide a brief description of three tropospheric zenith models. In addition, the estimation of the ionospheric zenith path delay is also described.

### 2.1. Simplified Static Zenith Model

In the preliminary test of the geolocation accuracy of GF-3, the atmospheric path delay is calculated by this model, which is consistent with the TerraSAR-X SAR products [3,9]. It can be written as follows:

(4) $$\Delta Z_{static}=\Delta Z_{sea}e^{-h/h_{0}}$$

where $\Delta Z_{sea}$ is the zenith delay at sea level, which amounts to about 2.3 m [11]. $h_{0}$ is the atmospheric thickness constant, and h is the height of the center of the scene. It is based on the static atmospheric parameter, so a meter-level error at most will be introduced to the geolocation results.

### 2.2. Analytic Approximation Zenith Model

These kinds of models are based on some representative meteorological data and approximate the meteorological changes in the zenith direction according to the surface meteorological parameters. Some analytic methods are used to calculate the parameters in the models. The representative models include the Saastamoinen (SAAS) model and the Hopfield model. The SAAS model is illustrated and verified in this paper for its popularity in GPS tropospheric path delay estimation.

The SAAS model divides the troposphere into two parts: The first layer ranges from the ground to the top of the troposphere at 12 km. In this layer, the atmospheric temperature decreases linearly with a decreasing rate of about 6.5 °C/km. The second layer ranges from the upper troposphere to the top of the stratosphere, and the temperature is a constant in this layer. The zenith path delay $\Delta Z_{SAAS}$ (m) can be calculated according to the surface meteorological parameters [19] as follows:

(5) $$\Delta Z_{SAAS}=0.002277\times \frac{\left(P_{s}+(0.05+\frac{1255}{T_{s}+273.15})e_{s}\right)}{f(\phi ,h)}$$

(6) $$e_{s}=rh\times 6.11\times 10^{\frac{7.5T_{s}}{T_{s}+273.15}}$$

where $T_{s}$ is the surface temperature (°C), $P_{s}$ is the surface pressure (mbar), $e_{s}$ is the surface vapor pressure, and rh is the relative humidity (0~1). f(ϕ,h) is a function of latitude ϕ and target height h (km) as follows:

(7) f(ϕ,h)=1−0.00266(2φ)−0.00028h.

### 2.3. Integral Zenith Model

This model is also referred to as the raytracing model in some papers [7,16]. Due to the inhomogeneity of the atmosphere, the atmospheric refraction varies with different altitudes. This model stratifies the atmosphere by height and calculates the path delay of each layer according to the different atmospheric refraction coefficients of each layer. Then, the total delay is the sum of the all layers’ path delays. The refractive index of each layer is based on the actual meteorological data. The greater the number of layers, the closer the total delay to the actual value. In this model, the tropospheric delay is usually divided into hydrostatic, wet, and liquid components. The liquid delay is too small to be considered. The atmospheric refraction index is used to calculate the delay in the nadir direction $\Delta Z_{\mathrm{int}}$ [20] as follows:

(8) $$\Delta Z_{\mathrm{int}}=10^{-6}\int_{s}Nd_{s}+\Delta Z_{top}$$

(9) $$N=N_{dry}+N_{wet}=k_{1}\frac{P}{T}+(k^{'}{}_{2}\frac{e}{T}+k_{3}\frac{e}{T^{2}})$$

where $N_{dry}$ and $N_{wet}$ represent the hydrostatic refractivity and wet refractivity, respectively. $\Delta Z_{top}$ is the tropospheric delay above the integral atmosphere. $k^{'}{}_{2}$ is written as follows:

(10) $$k^{'}{}_{2}=k_{2}-k_{1}\frac{R_{d}}{R_{w}}$$

$k_{1}=77.604K/\mathrm{mbar}$, $k_{2}=64.79K/\mathrm{mbar}$, and $k_{3}=377600K^{2}/\mathrm{mbar}$ are the atmospheric refraction constants. $R_{d}=287J/(K\cdot kg)$ is the dry gas constant, and $R_{w}=461J/(K\cdot kg)$ is the wet gas constant. The water vapor e pressure cannot be directly measured generally and can be derived by specific humidity q as follows:

(11) $$e=\frac{qP}{0.622+0.378q}.$$

Finally, the integral zenith delay is expressed as follows:

(12) $$\begin{array}{cc} \Delta Z_{\mathrm{int}}= & \Delta Z_{hdy}+\Delta Z_{wet}+\Delta Z_{top}=10^{-6}\int_{s}Nd_{s}+\Delta Z_{top}\\ & =10^{-6}\int_{s}(N_{hyd}+N_{wet})d_{s}+\Delta Z_{top}\\ & =10^{-6}(\sum_{i}N_{hdyi}\Delta s_{i}+\sum_{i}N_{weti}\Delta s_{i})+\Delta Z_{top} \end{array}.$$

### 2.4. Ionospheric Model

The ionospheric delay is inversely proportional to the square of the signal frequency. For the SAR signal, the ionospheric group delay is of interest, which can be calculated by the total number of electrons TEC on the propagation path as follows:

(13) $$\Delta Z_{iono}=\frac{40.28}{f^{2}\cos\theta }\int_{r}^{sv}N_{e}ds=\frac{40.28}{f^{2}\cos\theta }TEC$$

where the factor 1/cosθ is the map function, which converts the path delay from nadir to the path at a particular incidence angle θ. TEC denotes in units of 10^16^ (known as the total election content unit, TECU). Real TEC value can be acquired from global ionosphere maps (GIM), which are provided by the European Centre for Orbit Determination (CODE) every day. A total of 12 maps can be acquired every day because the GIM is updated every two hours [20]. The ionosphere has the biggest effect on the L-band and the smallest on the X-band in the SAR system. For GF-3, the center frequency of the C-band SAR instrument is 5.4 GHz. Given an incidence angle of 30° and a TEC of 10 TECU, the ionospheric delay is about 0.16 m. Although GF-3 operates at low earth orbit, the incomplete ionospheric delay is still significant for the geolocation accuracy.

## 3. Experiments and Results

In this section, two experiments will be carried out. In first part, the tropospheric zenith delays estimated by three tropospheric models are tested and analyzed. In second part, geometric calibration and accuracy evaluation of GF-3 are carried out using the three tropospheric models.

### 3.1. Analysis of Accuracy of Different Tropospheric Models

This experiment compares the zenith tropospheric delay (ZTD) measured from the atmosphere over two IGS stations with the value estimated by three tropospheric models. The meteorological data is acquired from ERA-Interim [21], which is the latest European Centre for Medium-Range Weather Forecasts (ECMWF) global atmospheric reanalysis of the period from 1978 to the present day. This data assimilation system includes a four-dimensional variational analysis (4D-Var) with a 12-h analysis window. The pressure levels data are generated four times (at 0, 6, 12, 18 h) per day with different spatial resolutions (0.125°~3°). It has a 37-vertical-layers range from 1 mbar to 1000 mbar corresponding to the height of 47 km. The weather data (geopotential, relative humidity, temperature, and specific humidity) at 6 h spanning from 1 January 2016 to 31 December 2016 was applied to the tropospheric model in the experiment.

**Figure 2:**
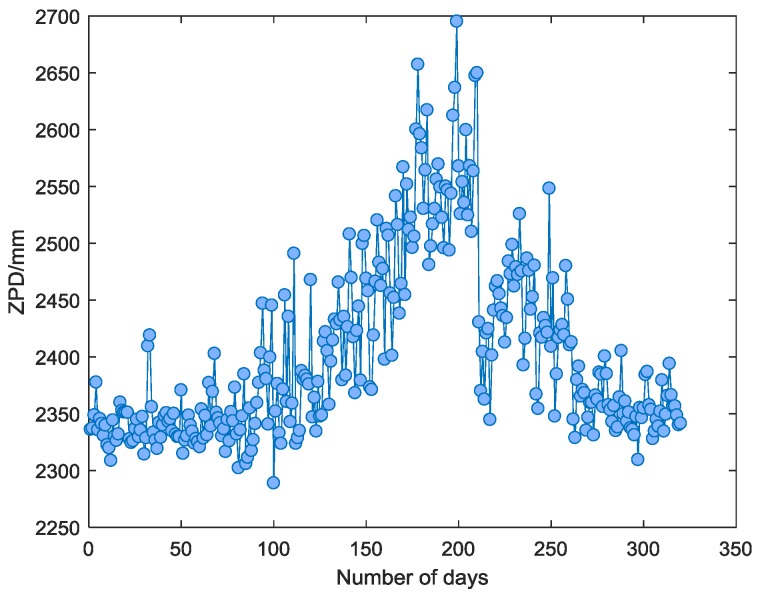
The zenith path delay (ZPD) variations at the BJFS International GNSS Service (IGS) station in 2016.

The International GNSS Service (IGS) is an international GNSS high-precision product community with three global data centers (Crustal Dynamics Data Information System, Scripps Orbit and Permanent Array Center, and Institut Geographique National), which provide free-range and phase observations, broadcast ephemeris, meteorology, and other measured data [22]. Since 1997, IGS has been providing zenith path delay (ZPD) products, which are updated every 5 min in each IGS station. The ZPD products in 2016 at the Beijing Fangshan (BJFS) IGS station (longitude: 115.89°, latitude: 39.61°, height: 87.41 m) are mainly used to contrast with the ZTD estimated by the tropospheric models. However, due to the lack of part of the data, a total of 321 days of ZPD data in BJFS (shown in Figure 2) is available. In addition, an extra experiment in the Urumqi (URUM) IGS station (longitude: 92.40°, latitude: 43.81°, height: 858.79 m) is carried out at the same time to verify the universality of the conclusion.

The meteorological data of the ECMWF and IGS ZPD products are fixed in the grid point, which is far from the IGS station; therefore, the zenith path delays at the IGS site need to be interpolated. The bilinear interpolation is used to calculate the accurate results according to the four grid points near the IGS station [23]. Furthermore, the top height of the pressure levels data is about 47 km (37th layer), and the part over 47 km is absent of meteorological data, which is essential to the integral model. In order to obtain the path delay above 47 km, the SAAS model combined with the top layer meteorological data (the 37th layer) is used to figure out the extra delay [18].

**Figure 3:**
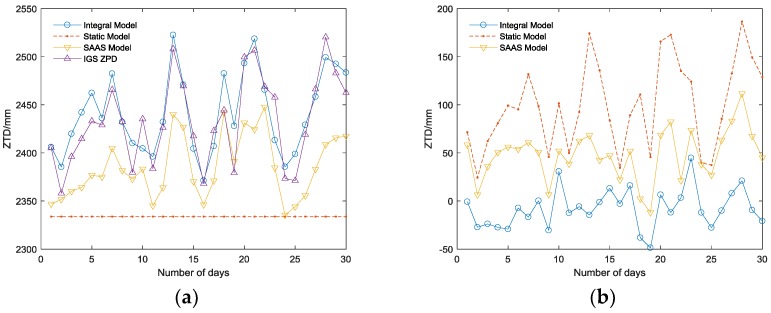
(**a**) The IGS ZPD variations and ZTD variations of the three models in June; (**b**) The difference value (DIFF) between ZTD of three models and IGS ZPD in June. SAAS: the Saastamoinen model.

**Figure 4:**
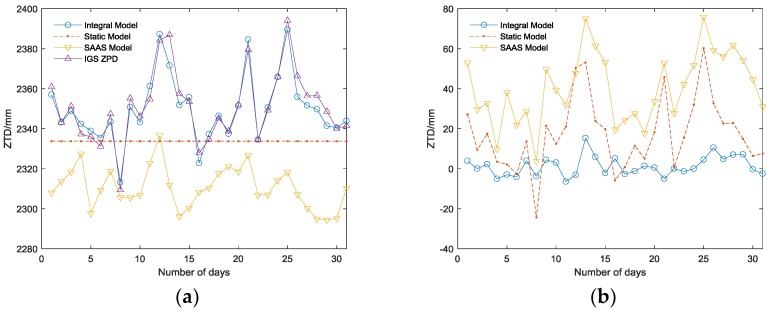
(**a**) The IGS ZPD variations and ZTD variations of the three models in December; (**b**) The DIFF between ZTD of three models and IGS ZPD in December.

Figure 3 and Figure 4 show the variation and difference value (DIFF) between ZTD estimated by three tropospheric models and the actual ZPD observed from the BJFS IGS station in two representative months: June and December.

**Figure 5:**
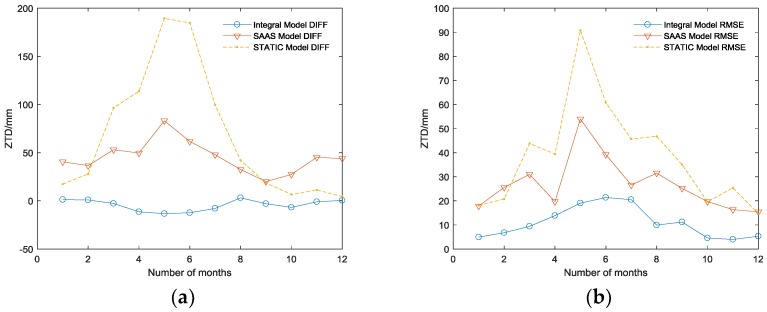
(**a**) The DIFF between ZTD of three models and IGS ZPD in 2016 (**b**) The RMSE between ZTD of three models and IGS ZPD in 2016.

Figure 5 shows the DIFF and the root-mean-square error (RMSE) between the estimated delay and the real delay of 12 months in 2016. The comparisons of mean DIFF and RMSE of the three models are listed in Table 1.

**Figure 6:**
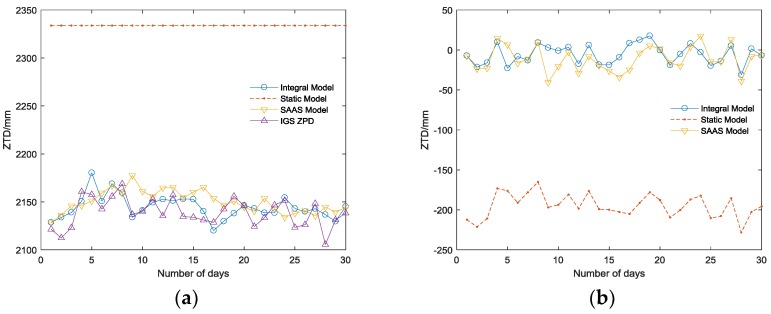
(**a**) The URUM IGS ZPD variations and ZTD variations of the three models in December (**b**) The DIFF between ZTD of three models and the URUM IGS ZPD in December.

The supplementary experiment (illustrated in Figure 6) in the URUM IGS station, which locates on a higher altitude (858.79 m) and has a dry atmosphere, is carried out to prove the universality of the conclusion.

Thus, the following can be concluded:

(1) Among the three tropospheric models, the integral model has the highest accuracy, which can reach the millimeter level (Table 1), and its RMSE is the smallest corresponding to the highest stability; the SAAS model has the second highest accuracy and can achieve an accuracy on the centimeter level in practice. The DIFF of the simplified static model is similar to that of the SAAS model in the BJFS IGS station, even though the fitting accuracy is better than the SAAS model in December. However, the experiment in the URUM IGS station proved (shown in Figure 6) that due to the lack of actual meteorological data, the universality of the simplified static model is very poor, and the actual fitting accuracy is far worse than the other two models, which are based on the real meteorological data.
(2) In terms of time, the DIFF and RMSE obviously have seasonal variation characteristics. Due to the high atmospheric water vapor, frequent precipitation, and changeable weather conditions in the summer, the DIFF and RMSE are obviously higher than that in the winter. As illustrated in Figure 3b, the highest RMSE of the integral model is no longer than 20 mm during the 12 months, which is much better than the other two models, verifying its excellent seasonal adaptability.
(3) In the area with less rainfall and less changeable weather, the simplified static model deployed in GF-3 can meet basic requirements; while in other area with complex terrain and changeable weather, there will be a large deviation in fitting path delay, and this model is unsuitable. Replacing the simplified static model with the integral model will increase the positioning accuracy in GF-3.

### 3.2. Assessment of GF-3’s Absolute Positioning Accuracy Using Different Tropospheric Models

In this part, the geolocation results were compared with or without different tropospheric models using GF-3 SAR imaging. Five scenes in different modes of GF-3 SAR images were chosen to verify the geolocation accuracy. The test areas included Songshan, Dalian, and Sanhe in China and Amazonas in Brazil. The data were collected from 28 January 2017 to 6 September 2017 in sliding spotlight (SL), fine strip 2 (FS2), standard strip (SS), and quad-polarization stripmap 2 (QPS2) mode. Details of the images are listed in Table 2. In this experiment, two contrastive experiments were carried out. The first compared the range accuracy updated by the integral model in same imaging date and different area. The second compared the range accuracy updated in same area and different imaging date.

The ground control points (GCPs) selected in the experiment were some natural targets (persistent scatterers, PSs) in cities that were manually extracted from Google Earth with a plane resolution of 0.5 m. Because the elevation in Google Earth is inaccurate with an error of over 20 m, test areas located in the seaside or plain were selected to eliminate the elevation errors. The actual elevation accuracy of the test sites was within 10 m. For SAR images, there was a positioning error within one pixel, because the oversampling process was unsuited to the determination of the position of the PSs. Moreover, the use of GF-3’s real-time orbit products in this experiment, which have a nominal accuracy of 10 m, will also lead to geolocation error.

Relying on the CODE ionospheric data and ECMWF tropospheric data, the atmospheric path delay, including the neutral atmospheric delay and ionospheric delay, were compensated by the different atmospheric models. The positioning accuracies of all GCPs in range are listed in Table 3. The updated last column in Table 3 is the improved range, replacing the static model with the integral model in the GF-3 geolocation procedure.

According to Table 3, an approximately 2~3 m error in range was introduced by the atmospheric path delay, and the integral model achieves the highest geolocation accuracy. The SAAS model also achieved close results, which were superior to the static model. Compared with the static model, the geolocation accuracy in range of the five tested images could be increased by an average of 0.547 m (FS2_1), 0.209 m (SL), 0.46 m (SS), 0.32 m (FS2_2), and 0.282 m (QPS2) by using the integral model.

In the first contrast test, the images of SL and FS2_1 were both obtained at 2017.9.6, while the SL image was acquired at Sanhe, which is an inland city in China, and the FS2_1 image was acquired at Dalian, which is a coastal city in China. It is obvious that updated range in Dalian was larger than that in Sanhe owing to the more changeable weather in the seaside. In the second test, the images were both taken from Songshan, China in January and July, respectively. The updated range in July was 0.038 m larger than that in January, proving the seasonal changes in tropospheric delay make a difference to the geolocation results. The range in the SS image acquired in March also had a relatively larger promotion, because the tropical Amazon rainforest has a large rainfall all year round.

Owing to the limited conditions, the natural targets in the city were selected as the GCPs in place of the corner reflectors. Although there existed elevation error, satellite trajectory error, and control points error, the positioning accuracy within 10 m was achieved in the experiment after the elimination of “start-stop approximation”, internal calibration delay, and atmospheric delay, which proves the good absolute positioning precision of GF-3 to natural ground targets.

The results of the experiments indicate that the integral model has a higher accuracy than the simplified static model, and it can improve the GF-3 geolocation accuracy effectively. In [3], a preliminary experiment of exploring the GF-3 absolute geolocation accuracy was conducted, and 2.56 m geolocation accuracy was reached using the simplified static model. It is predictable that the highest GF-3 absolute geolocation accuracy can be further promoted by close to 2 m by replacing the simplified static model with the integral model.

## 4. Summary and Outlook

This paper discusses the accuracy of different tropospheric models and the geolocation accuracy of GF-3 images using different models. Depending on the ECMWF tropospheric data and the IGS ZPD products, the accuracy of the integral model was proved to reach to the millimeter level. Compared with the simplified static model used in GF-3, the positioning error of several decimeters can be eliminated by the integral model.

According to the current research, there are still about 3 m geolocation errors in GF-3. It is estimated that the atmospheric error, SET error, and the corner reflector measurement error account for 1–2 m. In future work, a more thoughtful experiment will be carried out combined with the accurate atmospheric model and precise measured corner reflectors. It is believable that 1 m or even sub-meter geolocation accuracy will be achieved if these extra errors can be eliminated.

## Figures and Tables

**Table 1:** Comparisons of the mean DIFF(:mm) and RMSE(:mm) of the three models.

	DIFF_INT	RMSE_INT	DIFF_STATIC	RMSE_STATIC	DIFF_SAAS	RMSE_SAAS
**Mean**	−4.3807	10.908	67.6934	38.4067	45.1184	26.8379

**Table 2:** GaoFen-3 (GF-3) synthetic aperture radar (SAR) images with information regarding the test site.

Imaging Mode	Resolution	Date of Imaging	Imaging Region	Incidence Angle	Number of GCPs
**SL**	1	2017.9.6	Sanhe, China	29.67	6
**FS2_1**	10	2017.9.6	Dalian, China	30.62	6
**FS2_2**	10	2017.7.3	Songshan, China	42.3	4
**SS**	25	2017.3.19	Amazonas, Brazil	24.1	4
**QPS2**	25	2017.1.28	Songshan, China	37.31	5

**Table 3:** The geolocation accuracy in range of the three tropospheric models.

Mode	No Atmospheric Models (m)	Static Model (m)	SAAS Model (m)	Integral Model (m)	Updated (m) (Integral-Static)
**FS2_1**	10.378	8.024	7.589	7.475	0.549
	7.089	4.735	4.299	4.187	0.548
	5.436	3.086	2.646	2.544	0.542
	5.672	3.317	2.882	2.758	0.559
	8.244	5.89	5.454	5.348	0.542
	6.424	4.07	3.634	3.521	0.549
**SL**	7.844	5.172	5.026	4.968	0.204
	5.128	2.456	2.31	2.252	0.204
	4.726	2.054	1.909	1.835	0.219
	10.805	8.132	7.987	7.924	0.208
	8.757	6.084	5.939	5.86	0.224
	5.124	2.451	2.306	2.256	0.195
**SS0**	8.738	6.237	6.076	5.776	0.462
	8.564	6.063	5.903	5.612	0.451
	7.775	5.274	5.113	4.812	0.462
	3.95	1.449	1.289	0.984	0.465
**FS2_2**	5.296	2.089	1.866	1.761	0.328
	8.017	4.81	4.588	4.493	0.317
	10.644	7.437	7.213	7.139	0.298
	10.098	6.89	6.667	6.55	0.34
**QPS2**	4.243	1.347	1.086	1.068	0.279
	5.771	2.877	2.613	2.603	0.274
	8.146	5.25	4.986	4.962	0.288
	7.814	4.922	4.658	4.637	0.285
	7.003	4.108	3.844	3.824	0.284
